# Editorial

**DOI:** 10.4102/ajod.v6i0.423

**Published:** 2017-09-28

**Authors:** Jonathan Pearlman, Rory Cooper

**Affiliations:** 1 International Society of Wheelchair Professionals, University of Pittsburgh, United States; 2 Human Engineering Research Laboratories, Veterans Affairs Pittsburgh Healthcare System, United States; 3 Department of Rehabilitation Sciences and Technology, University of Pittsburgh, United States; 4 School of Health and Rehab Sciences, University of Pittsburgh, United States; 5 Department of Rehabilitation Science and Technology, Uniformed Services University of Health Sciences, United States

It is a pleasure to write this editorial for the special issue on Wheelchairs in Less-Resourced Settings in the *African Journal of Disability* (AJOD). We wish to thank the editorial staff of *AJOD* and specifically express our appreciation to Dr Leslie Swartz for the support. The manuscripts published as part of this special issue represent the most recent and some of the most important research being published to guide the wheelchair sector. The special issue was co-hosted by the International Society of Wheelchair Professionals (ISWP) which was launched in 2015. ISWP’s mission is to serve as a global resource for wheelchair service standards and provision through advocacy, training and testing, standards, evidence-based practice, innovation and a platform for information exchange. Supporting this special issue is one of the ways ISWP is working to encourage researchers, clinicians and policymakers to focus on the issues in the wheelchair sector and promote the evidence-based practice that can drive improvements in wheelchair service provision so that more people receive the high-quality affordable and appropriate wheelchairs that they need.

There have been impressive improvements that have helped to professionalise the wheelchair sector in less-resourced settings in the last 10 years, and the pace of those changes is increasing. A catalyst for this transformation occurred at a consensus conference in 2006 in Bangalore, India (Sheldon & Jacobs 2007), where stakeholders decided on a roadmap to strengthen the wheelchair sector, established the definition of an appropriate wheelchair and estimated the scale of the need (World Health Organization [WHO] 2011). This effort led to the publications from the WHO of guidelines on wheelchair provision in less-resourced settings (WHO 2008) and training resources (WHO 2016a, 2017a, 2017b). These documents, as well as other published training programmes (e.g. Coolen et al. 2004; Emergency Wheelchair Package 2017; Toro et al. 2017), have become important resources for organisations working in the wheelchair sector and governments working to serve their citizens under their obligations under the United Nations Convention on the Rights of People with Disabilities (UNCPRD) (UN 2006; WHO 2011). In spite of the progress, there is still a tremendous amount of work to be done to ensure wheelchair users have access to appropriate services and devices. The need is substantial – with over 75 million people in need of wheelchairs worldwide, and only 27% on average having access to them (WHO 2008).

A diverse group of organisations that take different approaches to provide wheelchairs are working to meet this tremendous need. Although the goal of providing reliable and safe mobility to individuals is similar, we know the outcome can vary significantly depending on the specific needs of the individuals and how well they are met by the products and services delivered. Comprehensive guidelines and training packages, such as those published by the WHO (2008, 2016a, 2017a, 2017b), help to establish a starting point to standardise wheelchairs services across the sector; however, robust research initiatives must be used to measure the outcomes of wheelchair services so that the strategies can be continually improved to achieve best practices. The WHO, through the Global Cooperation on Assistive Technology (GATE) (WHO 2016b) initiative, recently published a global research agenda for improved access to high-quality affordable assistive technology (WHO 2017c) that includes the following five research domains:

- effects, costs and economic impact of assistive technology
- assistive technology policies, systems, service provision models and best practices
- high-quality and affordable assistive technology
- human resources for the assistive technology sector
- standards and methodologies for the assessment of assistive technology need and unmet need.

The manuscripts published in this special issue are within these research areas and provide examples of the most recent evidence guiding the wheelchair sector in less-resourced settings. The effects of wheelchair services through different service provision models (domains 1 & 2) are described in Bazant et al. (2017), Shore (2017) and Ellapen et al. (2017). Policies (domain 2) related to accessibility to building infrastructure and higher education in Africa are covered in Yarfi, Ashigbi and Nakua (2017) and Chiwandire and Vincent (2017), respectively. The topic of high-quality and affordable wheelchairs (domain 3) is covered in several articles, including the works by Rispin, Huff and Wee (2017), Rispin, Hamm and Wee (2017) and Mhatre, Martin and McCambridge (2017) who describe a tool to measure the condition of wheelchairs, Mhatre, Ott and Pearlman (2017) who describe new standardised test methods to ensure wheelchairs are reliable, Stanfill and Jensen (2017) who describe field evaluation and Onguti et al. (2017) who describe a model for design competitions. Human resources for the assistive technology sector (domain 4) is the focus of several manuscripts, including the works by Fung et al. (2017) who describe an opportunity for integrating wheelchair services training into academic programmes worldwide, Norris (2017) who describes the benefits of peer training for wheelchair users and Munera et al. (2017) who describe the development of wheelchair services training of trainers programme being published by the WHO. The manuscript by Kamaraj et al. (2017) provides a conceptual framework for assessing the need and impact of wheelchair services provision based on a range of variables and fits within domain 5.

These manuscripts address important research questions that could lead to improvements in wheelchair service provision, and motivate additional research questions to be addressed in the challenging environments of less-resourced settings (Jefferds et al. 2011). A topic that stands out as one of the most important is in the study of effects, costs and economic impact of assistive technology (domain 1). Fundamental questions, such as the economic costs versus benefits of providing a wheelchair, remain unanswered. Among the researchers working in the wheelchair sector, this is not surprising because we view the need for wheelchairs through a human rights lens, as the human rights of people with disabilities are protected through national policies and codified in the Convention on the Rights of Persons with Disabilities (UN 2006). However, governments, especially their ministries of health, must be convinced to provide wheelchairs and require economic justifications. Not until a compelling economic case can be made to governments will mission-driven non-governmental organisations be able to scale to a global level to achieve our collective goal, and ISWP’s vision, that all people who need wheeled mobility devices receive the appropriate products and services with dignity be achieved.
